# A case report of agranulocytosis caused by acute hepatitis B virus infection

**DOI:** 10.1097/MD.0000000000047479

**Published:** 2026-01-30

**Authors:** Qian Liang, Kuanyin Tang, Wen Jiao, Yang Yuan, Xuxiu Lu, Hui Wang

**Affiliations:** a School of Clinical Medicine, Shandong Second Medical University, Weifang, Shandong, China; b Department of Infectious Diseases, Rizhao People's Hospital, Rizhao, Shandong, China; c Department of Central Laboratory, Rizhao People's Hospital, Rizhao, Shandong, China.

**Keywords:** acute hepatitis B virus infection, agranulocytosis, neutropenia

## Abstract

**Rationale:**

Acute hepatitis B virus (HBV) infection is clinically characterized by hepatic inflammation and parenchymal injury. As a systemic viral infection, HBV can reduce peripheral blood cell counts via suppressing bone marrow hematopoiesis. Agranulocytosis is defined as severe neutropenia, with an absolute neutrophil count below 0.5 × 10^9^/L. Standardized diagnostic and therapeutic protocols for HBV-associated agranulocytosis have not been established in clinical practice guidelines. Particularly, antiviral therapy in reversing HBV-induced bone marrow dysfunction remains to be fully elucidated. This case report aims to describe this clinical entity and elucidate the potential benefits of timely early antiviral intervention.

**Patient concerns:**

A 51-year-old female patient presented with unexplained fatigue persisting for over 20 days, coupled with anorexia and dysgeusia for greasy food.

**Diagnoses:**

Acute HBV infection complicated with neutropenia, confirmed by follow-up comprehensive evaluations after the initial laboratory findings of marked elevation of liver enzymes and leukopenia.

**Interventions:**

The patient received liver protection therapy with phosphatidylcholine and magnesium isoglycyrrhizinate, as well as supportive care to stimulate leukocyte production. Due to persistent leukopenia and detectable elevation of HBV deoxyribonucleic acid, entecavir was initiated for antiviral treatment.

**Outcomes:**

The above abnormal symptoms were relieved after antiviral therapy. Liver function and neutrophil counts returned to normal levels, and HBV deoxyribonucleic acid levels decreased significantly. Hepatitis B surface antigen seroconversion was achieved normal level after 6 months; therefore, clinical treatment was successfully discontinued. Follow-up showed sustained virologic and hematologic remission.

**Lessons:**

This case highlights acute HBV infection as a causative factor for severe neutropenia/agranulocytosis. Importantly, the study reveals that early antiviral treatment can not only suppress viral replication but also reverse bone marrow suppression, leading to complete hematologic recovery and functional cure (hepatitis B surface antigen seroconversion). This study demonstrates that antiviral therapy is necessary for the management of acute HBV with severe hematologic complications.

## 1. Introduction

Neutropenia refers to a group of syndromes characterized by an absolute neutrophil count (ANC) in peripheral blood of <1.5 × 10^9^/L. When the ANC falls below 0.5 × 10^9^/L, it is classified as severe neutropenia, or agranulocytosis, which is often accompanied by various types of infection. Its etiology can be self-limiting viral diseases or other diseases. At present, reports on agranulocytosis caused by acute hepatitis B virus (HBV) infection are rare. This paper reports a case in which leukopenia was the main reason for admission to the Department of Hematology. Further examinations suggested bone marrow suppression secondary to acute HBV infection, leading to agranulocytosis. After receiving antiviral therapy, liver-protective and enzyme-lowering treatments, as well as other supportive measures, her liver function and blood counts returned to normal, ultimately achieving clinical recovery. The diagnosis and treatment process of the patient was retrospectively analyzed and the literature was reviewed for the reference of clinicians.

## 2. Case presentation

A 51-year-old female patient was admitted to the hospital on April 26, 2024, due to “fatigue and general discomfort for more than 20 days.” Approximately 20 days prior, she developed fatigue and malaise without any obvious precipitating factors, accompanied by poor appetite, aversion to greasy food, and occasional nausea without vomiting. She did not initially seek medical attention or undergo any specific treatment. As her symptoms persisted without improvement, she visited a local hospital, where laboratory tests revealed the following: white blood cell count (WBC) 2.01 × 10^9^/L, neutrophil count 0.5 × 10^9^/L, ALT 427 U/L, AST 292 U/L, indicating abnormal liver function and leukopenia. For further evaluation, she was admitted to the Department of Hematology. She denied any history of chronic illness and had repeatedly tested negative for hepatitis B surface antigen.

Physical examination on admission: Body temperature: 36.5°C; heart rate: 78 beats/min; respiratory rate: 19 breaths/min; blood pressure: 125/80 mm Hg. The patient was alert and in good spirits, with no signs of anemia. There was no jaundice in the skin, mucosa, or sclera. No palpable superficial lymphadenopathy or sternal tenderness was detected. Cardiopulmonary examination revealed no significant abnormalities. The abdomen was soft with no tenderness or rebound pain. There was no percussion tenderness over the liver or kidney regions. The spleen was not palpable below the costal margin. No edema was noted in the lower extremities. Preliminary diagnoses: leukopenia; abnormal liver function.

**Figure 1.**
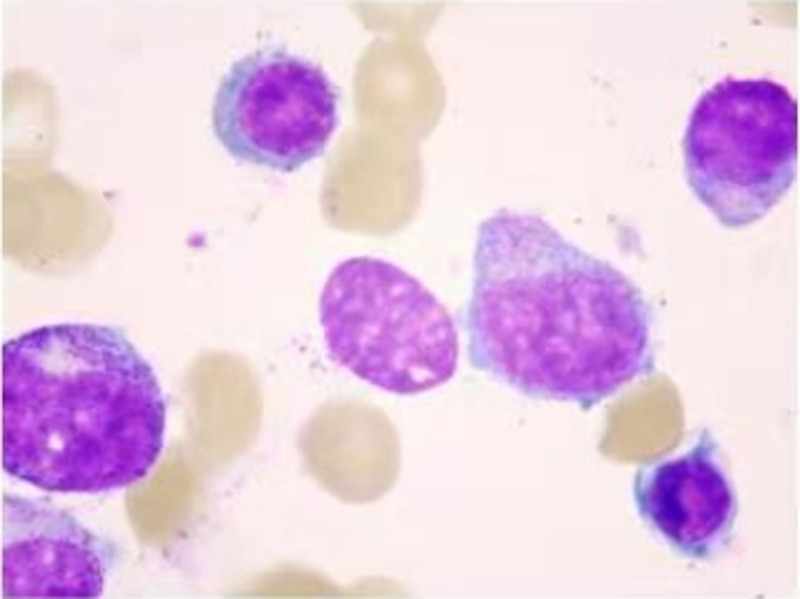
Representative images of bone marrow origin.

**Figure 2.**
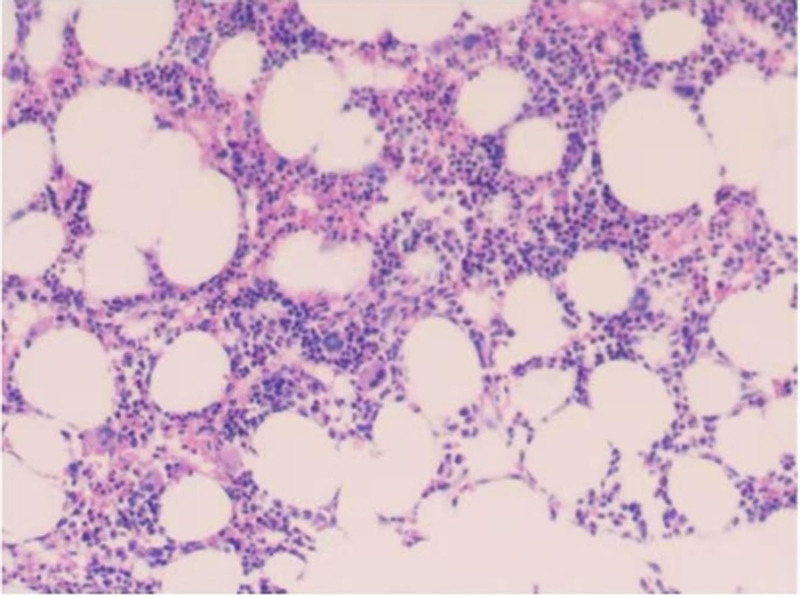
Bone marrow biopsy: HE staining: bone marrow hyperplasia is active, granulocyte, red and giant cell hyperplasia are possible (HE = Hematoxylin-Eosin).

After admission, on April 27, 2024, further tests were conducted, including coagulation profile, alpha-fetoprotein (AFP), tests for 4 common viral infections, thyroid function, antinuclear antibody spectrum, immunoglobulins and complement levels, and abdominal ultrasound, all of which were unremarkable. However, complete blood count and liver function remained abnormal (Table 1). HBV-DNA and hepatitis B serologic markers were positive (Table 2). Both the bone marrow smear (Fig. 1) and the peripheral blood smear showed findings consistent with leukopenia (Table 3). Bone marrow biopsy showed active hematopoiesis, with normal proliferation of granulocytic, erythroid, and megakaryocytic lineages (Fig. 2). Bone marrow karyotype analysis: 46, XX (20). Flow cytometry immunophenotyping: Excluded acute leukemia, lymphoma, plasma cell neoplasm, and high-risk MDS-related immunophenotypic abnormalities. Since no obvious abnormalities were seen in the bone marrow aspirate, the primary consideration was acute HBV infection. On April 27, 2024, the patient was transferred to the Department of Infectious Diseases for further treatment.

**Table 1:** Blood routine classification count and liver function changes.

Date	WBC (× 10^9^/L)	NEUT (× 10^9^/L)	LYMPH (× 10^9^/L)	ALT (U/L)	AST (U/L)	GGT (U/L)	ALP (U/L)
April 27, 2024	1.43	0.47	0.62	425	292	192	199
April 29, 2024	1.42	0.30	0.60	–	–	–	–
May 1, 2024	1.34	0.35	0.57	377	291	200	202
May 2, 2024	5.15	3.88	0.69	–	–	–	–
May 7, 2024	1.73	0.73	0.52	340	303	161	182
May 10, 2024	1.85	0.73	0.59	–	–	–	–
May 12, 2024	9.35	7.67	1.04	205	89	128	147
May 16, 2024	2.56	0.19	1.45	117	56	91	112
May 18, 2024	2.44	0.82	0.68	–	–	–	–
June 04, 2024	4.25	2.76	1.02	25	22	42	102

There was no significant abnormality in red blood cells, platelets and hemoglobin, which were not listed in the table. The patient was treated with human G-CSF on May 1, 2024 and May 10, 2024.

ALP = alkaline phosphatase, ALT = alanine aminotransferase, AST = aspartate aminotransferase, G-CSF = granulocyte colony-stimulating factor, GGT = gamma-glutamyl transferase, LYMPH = lymphocyte, NEUT = neutrophil, WBC = white blood cell.

**Table 2:** HBV serum markers and HBV-DNA changes.

Date	HBsAg (IU/mL)	Anti-HBs (IU/mL)	HBeAg (IU/mL)	Anti-HBe (IU/mL)	Anti-HBc (IU/mL)	HBV-DNA (IU/mL)
April 27, 2024	>250	<0.2	77.30	<0.048	35.2	26,600
April 30, 2024 (bone marrow)	–	–	–	–	–	4800
May 7, 2024	>250	0.82	18.4	<0.048	25.6	-
May 12, 2024	–	–	–	–	–	2770
May 18, 2024	2543 (COI)	<2 (COI)	8.4 (COI)	0.692 (COI)	0.011 (COI)	525
June 4, 2024	122	<0.2	0.055	0.240	10.8	35.5
October 24, 2024	0.37	7.98	0.1	0.07	0.1	–
January 8, 2025	0.16	90.3	0.05	0.1	0.1	<20

Anti-HBc = antibody to hepatitis B core antigen, Anti-HBe = antibody to hepatitis B e antigen, Anti-HBs = antibody to hepatitis B surface antigen, COI = cut-off index, HBeAg = hepatitis B e antigen, HBsAg = hepatitis B surface antigen, HBV = hepatitis B virus, HBV-DNA = hepatitis B virus-deoxyribonucleic acid.

**Table 3:** Blood smear and bone marrow smear granulocyte results.

Cell type		Blood smear	Bone marrow smear	
		%	Reference value	%
Promyelocyte	–	–	1.51–1.63	0.5
Neutrophil	Metamyelocyte	–	4.45–8.53	22
	Late myelocyte	–	5.93–9.87	12.5
	Band form	14	20.22–27.22	22
	Segmented form	22	6.52–12.36	6.5
Eosinophil	Metamyelocyte	–	0.15–0.61	–
	Late myelocyte	–	0.17–0.81	0.5
	Band form	1	0.64–1.86	–
	Segmented form	1	0.25–1.47	0.5
Basophil	Metamyelocyte	–	0.00–0.07	–
	Late myelocyte	–	0.00–0.13	–
	Band form	–	0.01–0.19	–
	Segmented form	3	0.00–0.08	–

The patient received symptomatic treatment, including polyene phosphatidylcholine and magnesium isoglycyrrhizinate injections for liver protection and enzyme reduction, along with agents to stimulate leukocyte production. On April 29, 2024, repeat blood tests showed further decreases in white blood cells and neutrophils. After discussion with the patient, antiviral therapy (entecavir) was initiated. On April 30, 2024, HBV-DNA (bone marrow) was 4800 IU/mL. The patient’s fatigue improved, and no fever or other discomforts were noted. Liver-protective and enzyme-lowering therapy, along with subcutaneous injections of granulocyte colony-stimulating factor (G-CSF), were continued. On May 12, 2024, HBV-DNA was rechecked and measured 2770 IU/mL, indicating a gradual decrease in viral load and suggesting that current treatment was effective. With improvement in fatigue, normalization of white blood cell count, and significant improvement in liver function, the patient was discharged in improved condition on May 18, 2024. HBV-DNA and hepatitis B serologic markers on May 18, 2024 are shown in Table 2. At outpatient follow-up on June 4, 2024 the patient’s complete blood count and liver function were normal, HBV-DNA levels had declined, but HBsAg remained positive (Table 2). After discharge, the patient continued oral entecavir antiviral therapy for 6 months and discontinued all medications (liver protection, leukocyte-boosting, and antiviral drugs) on October 24, 2024. As of the last follow-up on January 8, 2025, the results showed that blood routine and liver function indicators remained normal, HBsAg was negative, Anti-HBs was positive (90.3 IU/mL), and HBV-DNA was <20 IU/mL. No complications related to infection, liver injury, or hematological abnormalities occurred, and the patient achieved the criteria for clinical cure.

## 3. Discussion

Hepatitis B is primarily transmitted through vertical (mother-to-child) transmission, sexual contact, and iatrogenic exposure.^[1]^ Among these, acute HBV infections are mainly attributed to sexual transmission and medical exposure, though a significant proportion of cases (approximately 37.5%) have no clearly identified route of transmission. The incidence is higher in males than in females.^[2]^ Acute HBV infection is a common infectious disease worldwide, with clinical manifestations ranging from asymptomatic cases to nonspecific symptoms (such as fatigue, nausea, right upper abdominal discomfort, and jaundice), or even severe liver failure.^[1,3]^ Granulocytopenia caused by HBV infection is relatively rare, but in certain cases, it may exacerbate the patient’s condition and lead to more complex clinical presentations. This article presents a detailed report on the clinical diagnosis and treatment process of a patient with acute HBV infection complicated by severe granulocytopenia, aiming to reduce misdiagnosis and provide a reference for early and accurate clinical management.

Neutropenia is a syndrome characterized by an ANC in peripheral blood of <1.5 × 10^9^/L. When the ANC falls below 0.5 × 10^9^/L, it is classified as severe neutropenia, also known as agranulocytosis.^[^4^3]^ Neutropenia increases the risk of infection and also complicates treatment. Therefore, early identification and management of acute hepatitis B infection and its associated neutropenia are crucial for improving prognosis. In this case, the patient repeatedly exhibited an ANC of ≤0.5 × 10^9^/L, and oral leukocyte-boosting medications were ineffective, meeting the indication for treatment with G-CSF.^[4]^ After intermittent G-CSF administration, the patient’s fatigue and white blood cell levels showed significant improvement.

Traditionally, acute HBV infection is thought to primarily affect the liver, typically presenting with classic symptoms such as jaundice and abnormal liver function. A review of previously reported cases reveals that granulocytopenia associated with acute HBV infection is rarely documented. Iwamoto et a^[5]^l reported a case of a 29-year-old male who developed granulocytopenia approximately 2 months after the clinical onset of acute hepatitis B. Following treatment with G-CSF and lithium carbonate, the patient’s peripheral granulocyte count returned to normal. Brazille et al^[6]^documented a case of a patient with HIV who developed granulocytopenia 3 weeks after the onset of acute hepatitis B, which improved after treatment with hematopoietic growth factors. Similarly, in the present case, the patient developed granulocytopenia during the course of acute HBV infection, which improved after antiviral therapy and G-CSF administration, consistent with previously reported cases of acute hepatitis B-associated granulocytopenia. In prior reports, granulocytopenia typically occurred within 2 months of disease onset. In the current case, it appeared within 1 month of symptom onset, which aligns with the timeline observed in those earlier reports.

The mechanism by which acute hepatitis B infection leads to granulocytopenia remains unclear. Tseng et al^[7]^ suggested that natural killer cells and natural killer T cells may play a critical role in controlling early viral infection in acute hepatitis B by secreting interferon-γ (IFN-γ). Other studies have indicated that cytokines such as IFN-γ, TNF, IL-10, IL-6, and IL-4, along with CD8^+^ T lymphocytes and cytotoxic T lymphocytes, may be involved in viral clearance.^[8–12]^ These cytokines and immune cells are closely associated with inflammatory responses. The activation of the immune system and the inflammation triggered by the virus may impair bone marrow hematopoiesis,^[13]^ resulting in decreased white blood cell counts, particularly granulocytes. Some research has also suggested that HBV may directly infect hematopoietic stem cells in the bone marrow, affecting their proliferation.^[14,15]^ In this case, the patient experienced a sharp drop in ANC to ≤ 0.5 × 10^9^/L during the acute HBV infection window period. Comprehensive diagnostic evaluations excluded other common causes (such as EBV/CMV infection, drug toxicity, autoimmune diseases, and primary hematologic disorders), suggesting a direct association between HBV infection and granulocytopenia. After initiation of antiviral therapy with the nucleoside analogue entecavir, the patient’s HBV-DNA level declined (from 2.66 × 10^[4]^ IU/mL to below the detection limit), and neutrophil levels gradually recovered, which further supports a causal relationship. Although the present observations support the importance of antiviral therapy in reversing bone marrow suppression, this study has several limitations. As a single case report, the conclusion about the association between acute HBV infection and agranulocytosis cannot be generalized. In addition, the bone marrow HBV-DNA of this patient was not dynamically monitored. Additional observational studies or mechanism investigations are needed to identify potential avenues to better identify the best management strategies.

Acute HBV infection is generally considered self-limiting, and antiviral therapy is usually not required for patients without complications.^[1]^ However, in patients who develop granulocytopenia as a result of acute HBV infection, appropriate antiviral treatment – such as nucleos(t)ide reverse transcriptase inhibitors – should be selected based on the severity of the condition. Early initiation of antiviral therapy may help reduce the inflammatory response and alleviate bone marrow suppression. For patients with granulocytopenia, the appropriate use of G-CSF may promote granulocyte production and reduce the risk of infection. Given that neutropenia increases susceptibility to infections, infection prevention measures should be strengthened, and secondary infections should be detected early and managed promptly.

## 4. Conclusions

This case once again provides evidence supporting a potential association between HBV infection and granulocytopenia. For patients with acute hepatitis complicated by granulocytopenia, early initiation of antiviral therapy may help suppress viral replication, delay the progression of bone marrow suppression, and prevent secondary infections and other complications. It also highlights the importance for clinicians to closely monitor the immune system when managing HBV-related complications. In the future, larger-scale studies incorporating virological and immunological assessments are needed to further elucidate the molecular mechanisms by which HBV leads to granulocytopenia.

## Acknowledgments

The authors were genuinely grateful to People’s Hospital of Rizhao, for providing support for this case.

## Author contributions

**Conceptualization:** Hui Wang.

**Data curation:** Wen Jiao.

**Formal analysis:** Kuanyin Tang.

**Investigation:** Kuanyin Tang.

**Resources:** Xuxiu Lu.

**Supervision:** Hui Wang.

**Visualization:** Yang Yuan.

**Writing – original draft:** Qian Liang.

**Writing – review & editing:** Hui Wang.
